# Dichloroacetate treatment improves mitochondrial metabolism and reduces brain injury in neonatal mice

**DOI:** 10.18632/oncotarget.9150

**Published:** 2016-05-03

**Authors:** Yanyan Sun, Tao Li, Cuicui Xie, Yaodong Zhang, Kai Zhou, Xiaoyang Wang, Klas Blomgren, Changlian Zhu

**Affiliations:** ^1^ Department of Pediatrics, Third Affiliated Hospital of Zhengzhou University, Henan, China; ^2^ Center for Brain Repair and Rehabilitation, Institute of Neuroscience and Physiology, Sahlgrenska Academy, University of Gothenburg, Gothenburg, Sweden; ^3^ Zhengzhou Children's Hospital, Zhengzhou, China; ^4^ Department of Women's and Children's Health, Karolinska University Hospital, Karolinska Institutet, Stockholm, Sweden; ^5^ Perinatal Center, Institute of Neuroscience and Physiology, Sahlgrenska Academy, University of Gothenburg, Gothenburg, Sweden; ^6^ Henan Provincial Key Laboratory of Child Brain Injury, Zhengzhou, China

**Keywords:** apoptosis, autophagy, brain ischemia, mitochondria, pyruvate dehydrogenase, Pathology Section

## Abstract

The purpose of this study was to evaluate the effect of dichloroacetate (DCA) treatment for brain injury in neonatal mice after hypoxia ischemia (HI) and the possible molecular mechanisms behind this effect. Postnatal day 9 male mouse pups were subjected to unilateral HI, DCA was injected intraperitoneally immediately after HI, and an additional two doses were administered at 24 h intervals. The pups were sacrificed 72 h after HI. Brain injury, as indicated by infarction volume, was reduced by 54.2% from 10.8 ± 1.9 mm^3^ in the vehicle-only control group to 5.0 ± 1.0 mm^3^ in the DCA-treated group at 72 h after HI (*p* = 0.008). DCA treatment also significantly reduced subcortical white matter injury as indicated by myelin basic protein staining (*p* = 0.018). Apoptotic cell death in the cortex, as indicated by counting the cells that were positive for apoptosis-inducing factor (*p* = 0.018) and active caspase-3 (*p* = 0.021), was significantly reduced after DCA treatment. The pyruvate dehydrogenase activity and the amount of acetyl-CoA in mitochondria was significantly higher after DCA treatment and HI (*p* = 0.039, *p* = 0.024). In conclusion, DCA treatment reduced neonatal mouse brain injury after HI, and this appears to be related to the elevated activation of pyruvate dehydrogenase and subsequent increase in mitochondrial metabolism as well as reduced apoptotic cell death.

## INTRODUCTION

Perinatal asphyxia-induced hypoxic ischemic brain injury (hypoxic-ischemic encephalopathy, HIE) is an important cause of neonatal death, and it occurs much more often in developing countries [1]. Survivors of perinatal asphyxia are often left with developmental impairments such as mental retardation and cerebral palsy [2, 3]. Studies have revealed that secondary brain injury after hypoxia-ischemia (HI) is preceded by impairment of mitochondrial respiration, intra-mitochondrial accumulation of calcium, release of proapoptotic proteins from the mitochondrial intermembrane space, and downstream activation of caspase-9 and caspase-3 [4, 5]. Interventions against perinatal HI brain injury, such as hypothermia [6, 7] and erythropoietin [8, 9], have shown promising results in reducing the incidence of neurological disabilities. However, these treatments have only been applied to full-term infants and are not successful in all cases. There is a pressing need for a better understanding of the mechanisms of brain injury and for conducting comparative and translational studies on how to improve HI-induced metabolic dysfunction, reduce neuronal cell death, increase cell survival, and promote brain regeneration and repair after perinatal brain injury [10].

HI deprives the brain of oxygen and glucose, and this results in an initial depletion of ATP that leads to rapid energy failure and necrotic cell death. The adenylate energy charge is restored rapidly during reperfusion and re-oxygenation. This is followed by a later secondary energy failure that lasts for several days [11] and initiates a cascade of biochemical events that results in delayed apoptotic cell death and autophagic cell death [12, 13]. Persisting lactic acidosis has been shown to be associated with severe encephalopathy in neonatal HIE [14]. Therefore, treatments directed towards decreasing cerebral lactic acidosis and improving cerebral metabolism might ameliorate ischemic brain injury.

Dichloroacetate (DCA) is a small molecule inhibitor of pyruvate dehydrogenase kinase (PDK), which activates pyruvate dehydrogenase (PDH) and facilitates mitochondrial oxidation of glucose-derived pyruvate and lactate via the tricarboxylic acid cycle to produce ATP. DCA readily crosses blood brain barrier [15], and it has been used for decades to treat children with mitochondrial disorders characterized by lactic acidosis [16-18]. Long-term DCA administration is shown to be generally safe and well tolerated in pediatric patients [19, 20]. Previous studies found that DCA treatment improves the post-ischemic clearance rate of lactic acid and acidosis by activating the mitochondrial enzymes that transform pyruvate into acetyl-coenzyme A (AcCoA). This prevents the secondary energy failure [21], and the effect is more pronounced in the immature brain [22]. Furthermore, AcCoA is shown to be a metabolic master regulator of autophagy [23], and autophagic cell death contributes to HI brain injury [24]. Taken together, these previous studies indicate that DCA might have pronounced neuroprotective effects for neonatal HI brain injury. The purpose of this study was to investigate the effect of DCA treatment on brain injury in neonatal mice after HI.

## RESULTS

### DCA treatment reduced HI brain injury in neonatal mice

**Figure 1:**
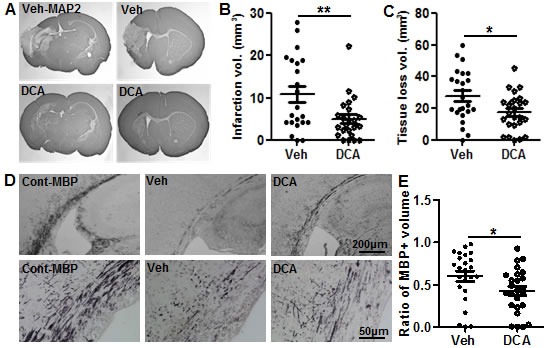
DCA treatment reduced brain injury after HI **A.** Representative MAP2 staining from the dorsal hippocampus (left panels) and striatum (right panels) at 72 h post-HI in vehicle-treated (upper panels) and DCA-treated mice (lower panels). **B.** The infarction volume at 72 h after HI in DCA-treated (*n* = 25) and vehicle-treated mice (*n* = 24). **C.** The total tissue loss volume at 72 h after HI in DCA-treated and vehicle-treated mice. **D.** Representative MBP staining at the hippocampal level shows the myelin structure in the subcortical white matter of the ipsilateral hemisphere at 72 h after HI in vehicle-treated and DCA-treated mice as well as in normal control mice. The lower panel in E shows higher magnification of MBP-stained subcortical white matter. **E.** Quantitative analysis showed the tissue loss in the subcortical white matter in DCA-treated (*n* = 25) and vehicle-treated mice (*n* = 24). *p < 0.05, **p < 0.01.

There were no treatment-related deaths. The body weight gain of the pups was from 4.43 ± 0.44 g at postnatal day (PND) 9 to 4.94 g ± 0.68 g at PND11 in the vehicle-treated group, and from 4.23 ± 0.70 g at PND9 to 4.98 g ± 0.89 g at PND11 in the DCA-treated group. HI-induced brain injury in neonatal mice was evaluated via immunohistochemistry staining for markers of gray matter (microtubule-associated protein 2, MAP2) and white matter (myelin basic protein, MBP) at 72 h after HI. The injury encompasses the cortex, hippocampus, striatum, and thalamus as indicated by the MAP2 staining. DCA treatment immediately after HI and then at 24 h and 48 h after HI was effective at reducing the severity of brain injury (Figure 1A). The gray matter injury, as indicated by the infarction volume calculated according to the measurement of positive MAP2 stained areas, was reduced by 54.2% from 10.8 mm^3^ ± 1.9 mm^3^ in the vehicle-treated mice to 5.0 mm^3^ ± 1.0 mm^3^ in the DCA-treated mice (*p* = 0.008) (Figure 1B). The overall volume of brain tissue loss was reduced by 37.2% in DCA-treated mice compared to vehicle-treated mice (*p* = 0.037) (Figure 1C). Myelination was visualized in the sub-cortex by MBP staining at PND 12, and the subcortical white matter displayed abnormal myelin structure in the brain hemisphere that is ipsilateral to the injury (Figure 1D). DCA treatment reduced the HI-induced decrease in the MBP-positive volume in the subcortical white matter by 29.1% (*p* = 0.018) compared with vehicle-treated mice (Figure 1E).

### DCA enhanced mitochondrial metabolism after HI in the neonatal mouse brain

**Figure 2:**
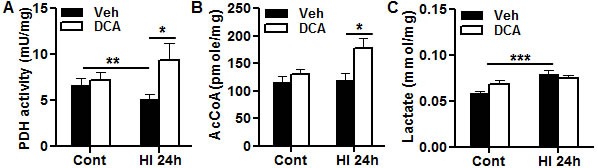
Effect of DCA treatment on brain mitochondrial metabolism Bar graphs show the effects of DCA treatment on control (Cont) at PND10 and at 24 h post-HI. **A.** Pyruvate dehydrogenase (PDH) activity assays in the brain cortical mitochondrial fraction. **B.** Acetyl-coenzyme A (AcCoA) concentrations in the brain cortical mitochondrial fraction. **C.** Lactate concentration in the brain cortical total homogenate. For all three assays, n = 8 for vehicle group, *n* = 7 for DCA group.*p < 0.05, **p < 0.01, *** *p* < 0.001.

PDH activity was measured 24 h after HI in the brain cortical mitochondrial fraction in vehicle-treated and DCA-treated mice. PDH activity decreased significantly at 24 h after HI compared with that of non-HI controls in the vehicle-treated groups (PND10) (*p* = 0.0037), and DCA treatment prevented the PDH activity decline at 24 h after HI compared with vehicle-treated mice (*p* = 0.0396) (Figure 2A). As a result, AcCoA in the DCA-treated group increased significantly in the mitochondrial fraction compared with the vehicle-treated groups at 24 h after HI (*p* = 0.024) (Figure 2B). Lactate was also measured at 24 h after HI in the cortical homogenate, and lactate increased significantly at 24 h after HI compared with that of non-HI controls in the vehicle-treated groups (*p* = 0.0002) (Figure 2C).

### Effect of DCA treatment on mitochondrial biogenesis in the neonatal mouse brain after HI

**Figure 3:**
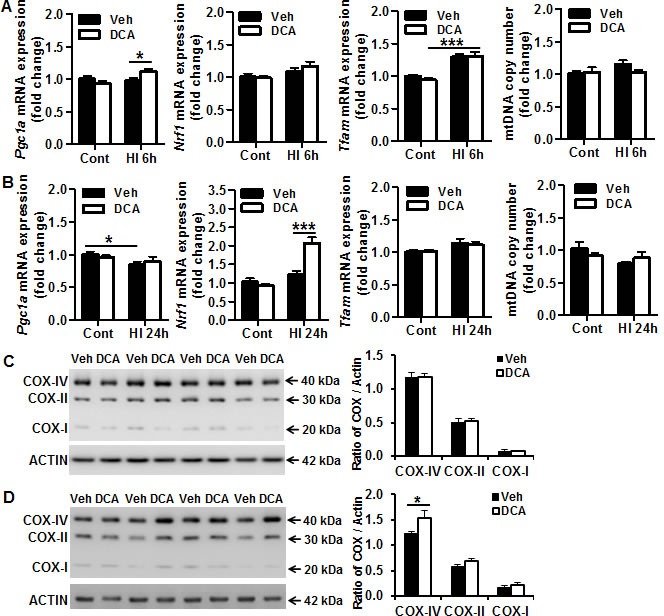
Effect of DCA treatment on brain mitochondrial biogenesis **A.** mRNA expression of mitochondrial biogenesis-related genes (*Pgc1α, Nrf1, Tfam*) and mtDNA copy number (mtDNA) in DCA-treated and vehicle-treated mice at 6 h after HI. **B.** mRNA expression of mitochondrial biogenesis-related genes (*Pgc1α, Nrf1, Tfam*) and mtDNA copy number (mtDNA) in DCA-treated and vehicle-treated mice at 24 h after HI. For all of the above assays, *n* = 11 for the vehicle control, *n* = 13 for the DCA control, *n* = 6 for the 6 h HI and 6 h DCA groups, *n* = 8 for the 24 h HI vehicle group, and *n* = 7 for the 24 h HI DCA group. **C.** Immunoblotting of COX-I, II, and IV in the mitochondrial fraction of normal controls treated with vehicle (Veh) or DCA. **D.** Immunoblotting of COX-I, II, and IV in the mitochondrial fraction at 24 h after HI showing that and DCA treatment increased COX-I, II, IV expression, and this was more pronounced with COX-IV. *p < 0.05, ***p < 0.001.

To determine if DCA treatment has any effect on mitochondrial biogenesis, the brain mRNA expression levels of peroxisome proliferator-activated receptor γ coactivator-1α (*Pgc1α*, which plays a key role in mitochondrial biogenesis and energy metabolism), mitochondrial transcription factor A (*Tfam,* which is a key activator of mitochondrial transcription and is a participant in mitochondrial genome replication), and nuclear respiratory factor 1 (*Nrf1,* which functions as a transcription factor that activates some genes regulating cellular growth and mitochondrial respiration) were examined by RT-PCR at 6 h and 24 h after HI in the vehicle and the DCA treatment group (Figure 3A, 3B). *Pgc-1α* mRNA expression in the neonatal mouse brain was not significantly changed after HI compared with non-HI controls at 6 h, but it decreased at 24 h after HI (*p* = 0.0152) (Figure 3B). DCA treatment increased *Pgc-1α* mRNA expression significantly at 6 h after HI compared with the vehicle treatment group (*p* = 0.034). *Nrf1* mRNA levels in the mouse brain did not begin to increase until 24 h (*p* = 0.001) after HI in the DCA-treated group compared with the vehicle-treated group (Figure 3B). *Tfam* mRNA expression was significantly increased at 6 h after HI (*p* < 0.001), and DCA treatment had no significant effect on *Tfam* mRNA expression (Figure 3A). We checked the transcription of mitochondrial genes (mtDNA), and no significant change was observed regardless of whether or not the pups were treated with DCA or subjected to HI (Figure 3A, 3B). We further checked the mitochondria-encoded cytochrome c oxidase (COX) subunits I, II, and IV in the mitochondrial fraction of normal controls (Figure 3C) and at 24 h after HI (Figure 3D). DCA treatment increased COX-IV at 24 h after HI (*p* = 0.016), but not COX-I and COX-II. The expression of these proteins did not change in the uninjured controls after DCA treatment.

### Effect of DCA treatment on mitochondrial fission and fusion in the neonatal mouse brain after HI

**Figure 4:**
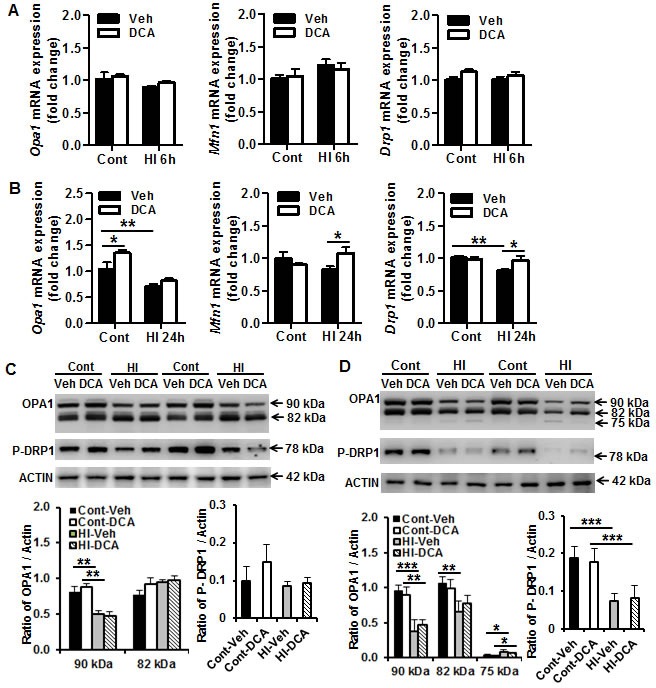
Effect of DCA treatment on brain mitochondrial fission and fusion **A.** The mRNA expression levels of the mitochondrial fusion (*Mfn1, OPA1*) and fission (*Drp1*) genes in the mouse brain were quantified by RT-qPCR at 6 h after HI. **B.** The mRNA expression levels of the mitochondrial fusion (*Mfn1, OPA1*) and fission (*Drp1*) genes in the mouse brain were quantified by RT-qPCR at 24 h after HI. *n* = 5 for the vehicle control, *n* = 6 for the DCA control at 6 h, *n* = 6 for the 6 h HI and 6 h DCA groups; *n* = 6 for the vehicle control, *n* = 7 for the DCA control at 24 h, *n* = 8 for the 24 h HI vehicle group, and *n* = 7 for the 24 h HI DCA group. **C.** Immunoblotting of OPA1, MFN1, and P-DRP1 in the mitochondrial fraction of normal control (Cont) and 6 h after HI treated with vehicle (Veh) or DCA. The 90 kDa upper band of OPA1 was decreased significantly at 6 h after HI, and DCA treatment did not prevent the reduction (*n* = 6/group). **D.** Immunobloting of OPA1, and P-DRP1 in normal controls and 24 h after HI treated with vehicle or DCA. The expression of these proteins decreased significantly at 24 h after HI in the mitochondrial fraction. The 75 kDa cleavage band of OPA1 increased significantly after HI, and DCA treatment has no significant effect on OPA1 cleavage at 24 h after HI (*n* = 6/group). *p < 0.05, **p < 0.01.

To examine the HI-induced changes in mitochondrial dynamics in the neonatal mouse brain and the possible influence of DCA treatment on these changes, we examined the transcription of the optic atrophy 1 (*Opa1*) and mitofusion (*Mfn1*) fusion genes and the dynamin-1-like protein (*Drp1*) fission gene as well as their protein expression in mitochondria at different time points after HI (Figure 4). There were no significant changes in these genes at 6 h after HI compared with the controls (Figure 4A), but there was decreased transcription of *Opa1* (*p* = 0.009) and *Drp1* (*p* = 0.001) at 24 h after HI. DCA treatment prevented HI-induced reduction of *Mfn1* (*p* = 0.033) and *Drp1* (*p* = 0.011) at 24 h after HI (Figure 4B). We further checked the protein expression of these genes and found that OPA1 and phospho-DRP1 (Ser637) (P-DRP1) all began to decrease at 6 h after HI (Figure 4C) and continued to decrease at 24 h after HI. The cleavage product of OPA1 increased significantly at 24 h after HI (*p* = 0.024). The increased cleavage product of OPA1 indicates that mitochondrial fusion was reduced, and the decrease in P-DRP1 reduces inhibition on mitochondrial fission and indicates that HI insult caused an increase in mitochondrial fission. However, DCA treatment did not prevent HI-induced reduction of P-DRP1 or cleavage of OPA1 in mitochondria (Figure 4D).

### DCA treatment reduced autophagy in neonatal mouse brain after HI

**Figure 5:**
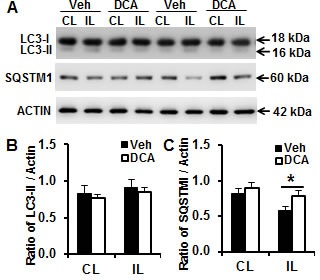
DCA treatment reduced autophagy activity in the mouse brain after HI **A.** Representative immunoblotting pictures show the expression of LC3 and SQSTM1 in the brain mitochondrial fraction at 24 h after HI. **B.** Quantification of LC3-II immunoblotting normalized to actin in the ipsilateral hemisphere (IL) compared to the contralateral hemisphere (CL) in the vehicle and DCA treatment group. **C.** Quantification of SQSTM1 immunoblotting in the ipsilateral hemisphere in the vehicle and DCA treatment groups. *n* = 7 for the vehicle group and *n* = 6 for the DCA group. *p < 0.05.

Autophagic cell death is another important contribution to neonatal brain injury [24, 25]. Autophagy activity was detected by immunoblotting for microtubule-associated protein 1 light chain 3 (LC3)-II and SQSTM1/p62 (a protein known to be selectively degraded by autophagy) in the brain cortical mitochondrial fraction at 24 h after HI (Figure 5A). The quantification of the LC3-II band (16 kDa) did not show a significant increase in the ipsilateral brain hemisphere compared to the contralateral brain hemisphere, and no difference between vehicle and DCA treatment groups was detected (Figure 5B). SQSTM, however, decreased in the ipsilateral brain hemisphere compared to the contralateral hemisphere in the vehicle-treated mice, and DCA treatment prevented the HI-induced SQSTM decrease compared to the vehicle-treated group (*p* = 0.041) (Figure 5C). This provides indirect evidence that DCA treatment reduces autophagy activity in the mouse brain after HI.

### DCA treatment reduced caspase-independent apoptotic cell death in the neonatal mouse brain after HI

**Figure 6:**
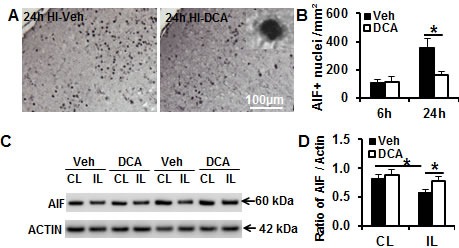
DCA treatment reduced AIF nuclear translocation in the mouse brain after HI **A.** Representative AIF staining in the cortex at 24 h after HI. **B.** AIF-positive nuclei were reduced in the border zone of the cortical infarction in the DCA-treated group and the vehicle-treated group at 24 h after HI (*n* = 8 for the vehicle group, *n* = 7 for the DCA group). **C.** Representative pictures show immunoblotting of the mouse brain mitochondrial fraction at 24 h after HI from the contralateral hemisphere (CL) and ipsilateral hemisphere (IL) in both vehicle (Veh) and DCA treatment groups. **D.** Quantification of AIF immunoblotting normalized to actin in the vehicle treatment group and DCA treatment group. n = 7 for the vehicle group and *n* = 6 for the DCA group. *p < 0.05.

Compared to the adult mouse, both the apoptosis-inducing factor (AIF)-dependent and AIF-independent apoptotic cell death pathways are more pronounced in the immature brain in HI-induced brain injury [26]. Caspase-independent apoptotic cell death, as indicated by AIF nuclear translocation, was investigated in the cortex in the mouse brain. AIF nuclear translocation was increased at the examined time points after HI (Figure 6A), and DCA treatment reduced AIF release from the mitochondria and translocation to the nucleus in the mouse brain cortex region at 24 h after HI, which was when caspase-independent apoptotic cell death reached its peak level (*p* = 0.009) (Figure 6B). Immunoblotting of the brain mitochondrial fraction at 24 h after HI showed reduced AIF in the ipsilateral hemisphere compared to the contralateral hemisphere (Figure 6C), and this was more pronounced in the vehicle treatment group than the DCA treatment group (25.9% vs. 12.7%, respectively) (Figure 6D).

### DCA treatment reduced caspase-dependent apoptotic cell death in the neonatal mouse brain after HI

**Figure 7:**
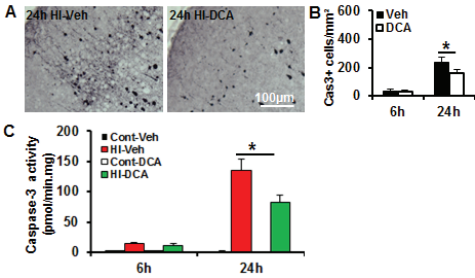
DCA treatment decreased caspase-3 activation in the mouse brain after HI **A.** Representative pictures show staining for the active form of caspase-3 in the brain cortex at 24 h after HI. **B.** Quantification of the caspase-3-positive cells in the IHC in the cortex. **C.** The caspase-3 activity (as indicated by DEVD cleavage) was assayed in the brain cortical homogenate at 6 h and 24 h after HI in the DCA-treated mice (*n* = 7) and the vehicle-treated mice (*n* = 8). *p < 0.05.

Caspase-dependent apoptotic cell death was investigated by applying immunostaining for the active form of caspase-3 in the injured brain areas after HI in PND9 mice (Figure 7A). DCA treatment reduced caspase-3 activation in the cortex at 24 h (*p* = 0.021) after HI - when the apoptotic cell death reaches its peak [27] - compared to the vehicle treatment group (Figure 7B). As a sensitive marker of caspase-dependent apoptosis cell death, caspase-3 activity was increased as early as 6 h after HI and reached its highest level at 24 h after HI. DCA treatment significantly reduced caspase-3 activation at 24 h (*p* = 0.021) after HI compared to the vehicle treatment group (Figure 7C).

## DISCUSSION

DCA has been used safely for decades to reduce cerebral lactate in children with genetic mitochondrial diseases [20], and it has been shown to resolve cerebral lactic acidosis to a greater degree in the immature brain [22]. Experimental studies have shown that DCA treatment also prevents secondary energy failure at early reperfusion after cerebral ischemia [28] and that it reduces ischemic brain injury in adult rats [29]; however, it is unknown whether or not it is also neuroprotective in HI-induced neonatal brain injury in rodents. In this study, we show for the first time that DCA treatment after HI increases mitochondrial PDH activity and AcCoA production, decreases autophagy and apoptotic neuronal cell death, and has a neuroprotective effect in neonatal mice.

The tricarboxylic acid cycle is a series of chemical reactions that take place in mitochondria to generate energy in the form of ATP. As part of this cycle, PDH mediates AcCoA formation from pyruvate, which feeds the electron transport chain responsible for ATP synthesis and oxygen consumption. Phosphorylation of PDH by PDK inactivates PDH, and this promotes pyruvate conversion to lactate instead of AcCoA in glycolysis and thus limits the entry of pyruvate into the mitochondria. DCA inhibits PDK, and this means that most of the PDH will be in the active form and thus pyruvate will move into the mitochondria for conversion to AcCoA and the subsequent generation of citrate and ATP. HI insult induces mitochondrial impairment as well as primary and secondary phases of energy failure and cell death [30]. In the present study, we found that DCA treatment immediately after HI increased AcCoA and reduced neuronal cell death and brain injury. Thus the neuroprotective effect of DCA might be at least in part related to increased PDH activity, which increases the cerebral metabolic rate after the HI insult.

Mitochondrial biogenesis as part of normal cellular respiration, and mitochondrial fusion and fission for maintenance of organelle function, are crucial for functional recovery of neurons after injury, and this is a highly regulated process that requires the participation of both the nuclear and mitochondrial genomes [31]. Cerebral insults cause mitochondrial dysfunction, which is reflected by mtDNA damage and changes in mtDNA content, as well as changes in the transcription of mitochondrial genes and nuclear genes encoding mitochondrial proteins, changes in protein synthesis, and changes in mitochondrial function. *PGC-1α* serves as a key regulator of energy metabolism, and it can co-activate numerous metabolically relevant nuclear and non-nuclear receptor transcription factors such as *Nrf-1* and *Tfam* [32]. DCA treatment increased mitochondrial biogenesis-related *Pgc-1α* mRNA at 6 h and *Nrf-1* mRNA at 24 h after HI, but not *Tfam* mRNA. The different expression profiles of these genes might be related to different temporal changes after HI [32, 33]. Even though one of the mitochondria-specific proteins increased at 24 h after HI with DCA treatment, there was no change in mtDNA copy number in the normal controls at either 6 h or 24 h after HI. Furthermore, DCA treatment had no effect on mitochondria-specific proteins in the normal controls. These results suggest that DCA treatment alters some of the mitochondrial biogenesis-related genes, but it appears to have no significant effect on mitochondrial biogenesis in the neonatal mouse brain after HI.

Mitochondria are highly dynamic organelles that undergo permanent fusion and fission, an antagonistic process that links mitochondrial dynamics to the balance between energy demand and nutrient supply [34] and is important for mitochondrial function and cellular survival [35]. Highly fused mitochondria are formed under nutrient deprivation to optimize mitochondrial function and to maximize ATP synthesis. In the pathological condition of HI, maintaining mitochondrial fusion might have a protective effect. In contrast, mitochondrial fission is activated under conditions of excess nutrients [36]. In the current study, HI insult decreased the expression of the mitochondrial fusion gene *Opa1*, and there was a concomitant decrease in expression and increase in cleavage of the OPA1 protein. These data suggest that HI insult induces a reduction in mitochondrial fusion. Because phosphorylation at Ser637 of DRP1 inhibits mitochondrial fission, the downregulation of P-DRP1 observed after HI insult indicates that HI insult leads to increased mitochondrial fission. These data are consistent with previous reports that ischemia reperfusion induces an increase of mitochondrial fission [37-39]. However, even though DCA treatment altered mitochondrial fusion and fission-related genes, it did not affect the expression of P-DRP1 or cleavage of OPA1. These results suggest that neuroprotection by DCA treatment in the current study was not related to mitochondrial fusion or fission.

Autophagy is an important physiological mechanism to maintain cellular homeostasis and survival, and the immature brain retains its autophagic machinery to a greater extent than the adult brain [26] as part of normal brain development [40, 41]. Thus, basal autophagy plays an essential role in cell survival under physiological conditions. However, increased autophagy can be activated by many forms of stress such as starvation and ischemic insult [42-44]. Starvation or nutrient depletion results in the depletion of intracellular AcCoA, which is one of the physiological triggers of autophagy to recycle amino acids and fatty acids to produce energy and remove damaged organelles. In the immature brain, energy failure is induced rapidly after HI [11], and autophagy activity - as indicated by the autophagosome-related marker LC3-II - increases [26]. Inhibition of autophagy provides neuroprotection [24, 45]. These results indicate that autophagy could be considered a potential therapeutic target for the treatment of perinatal HI brain injury. In this study, DCA administration increased AcCoA production and reduced autophagy activity - as indicated by the sustained levels of SQSTM1 - and these effects might at least partly contribute to neuroprotection.

Apoptosis plays a more prominent role in the immature brain [26], and this includes both caspase-dependent and -independent pathways, and combined inhibition of both pathways provides synergistic protection against neonatal HI brain injury [46]. DCA treatment inhibits mitochondrial release of AIF, and although the precise mechanism is not clear, this process appears to be related to improved mitochondrial energy metabolism and reduced mitochondrial membrane permeability at early stages after the injury. This reduced permeability leads to a reduction in the amount of pro-apoptotic proteins, such as AIF and cytochrome c, that are released from the mitochondrial intermembrane space after the insult. Cytochrome c release from the mitochondria into the cytosol leads to activation of procaspase-9, which in turn cleaves and activates procaspase-3 [47] and leads to apoptosis. These data suggest that DCA-mediated neuroprotection in the neonatal mice might involve multiple anti-apoptotic mechanisms.

DCA as an orphan drug has been used for decades to treat children with inborn errors of mitochondrial metabolism, and it has been shown to have beneficial effects [17, 19] with higher doses being more effective [48]. DCA is neuroprotective at a dose of 100 mg/kg, but not at a lower dose of 10 mg/kg [28, 29]. Long-term DCA administration is considered generally safe and well tolerated in pediatric patients [19, 20]. It has been reported that daily DCA treatment at 25 mg/kg is associated with reversible neuropathy toxicity [49], but it cannot be determined whether this is attributed to DCA or to progression of underlying disease [20]. This inconsistency might be related to different conditions in the patients. In neonatal HI with an acute mitochondrial metabolic disturbance, DCA treatment improves mitochondrial metabolism over the short term, which is different from hereditary defects in mitochondrial metabolism that require long-term treatment.

In summary, post-ischemic DCA treatment reduces both gray-matter and white-matter injury in neonatal mice after HI insult. DCA treatment could therefore be a useful therapeutic strategy to improve neurological outcomes in perinatal asphyxia-induced brain injury.

## MATERIALS AND METHODS

### Cerebral hypoxia-ischemia

PND 9 C57BL/6J male mouse pups were purchased from Charles River (Germany) and housed with their dams in a temperature-controlled and pathogen-free environment with a 12:12-hour light-dark cycle [50, 51]. The pups were randomly assigned to DCA or phosphate-buffered saline (PBS) treatment groups. The pups were anesthetized with isoflurane (5% for induction, 1.5-2.0% for maintenance) in a mixture of nitrous oxide and oxygen (1:1). The left common carotid artery was isolated and cut between double ligatures of prolene sutures (6.0). The duration of anesthesia was less than 5 min. After the surgical procedures, the wounds were infiltrated with lidocaine for analgesia, and the pups were returned to their cages with the dams and allowed to recover for 1 hour. The pups were then placed in an incubator perfused with a humidified gas mixture (10% ± 0.01% oxygen in nitrogen) at 36°C for 40 minutes to induce a mild to moderate brain injury. After HI, the pups were returned to their dams until they were sacrificed. Control pups were not subjected to ligation or hypoxia. All animal experiments were approved by the Animal Ethical Committee of the University of Gothenburg (112 /2014).

### DCA administration

The DCA (10 mg/mL dissolved in PBS, DCA-lab, Lithuania, 2156-56-1) was prepared freshly prior to use and injected intraperitoneally at a dose of 100 mg/kg immediately after HI. Two additional injections (100 mg/kg) were administered at 24-hour intervals, and the pups were sacrificed at 72 h after HI. Control pups received an equivalent volume of PBS.

### Immunohistochemistry staining

At 6 h, 24 h, and 72 h after HI-induced injury, the pups were deeply anesthetized with 50 mg/ml phenobarbital and perfused intracardially with PBS and 5% buffered formaldehyde (Histofix; Histolab, Gothenburg, Sweden). Brains were removed and fixed in 5% buffered formaldehyde at 4°C for 18-24 h. After dehydration with graded ethanol and xylene, the brains were paraffin-embedded and cut into 5 μm coronal sections. Every 100th section throughout the whole brain for microtubule-associated protein 2 (MAP2) staining and myelin basic protein (MBP) staining (DCA = 25, vehicle = 24, 72 h), and every 50th section in the hippocampus for caspase 3 staining (DCA = 7, vehicle = 8, 24 h), were deparaffinized in xylene and rehydrated in graded ethanol concentrations. Antigen retrieval was performed by heating the sections in 10 mM boiling sodium citrate buffer (pH 6.0) for 10 min. Nonspecific binding was blocked for 30 min with 4% donkey or goat serum in PBS for 30 min. The primary antibodies were monoclonal mouse anti-MAP2 (1:1000 dilution, clone HM-2, Sigma, M9942), rabbit anti-MBP (1:10,000 dilution, Sternberger Monoclonal Incorporated, SMI 94), rabbit anti-active caspase-3 (1:150 dilution, BD Pharmingen, 559565), and goat anti-AIF (1:100 dilution, 2 μg/ml, Santa Cruz Biotechnologies, sc-9416). After incubating the sections with the primary antibodies at 20°C for 60 min or overnight at 4°C, the appropriate biotinylated secondary antibodies (1:200 dilution; all from Vector Laboratories, Burlingame, CA, USA) were added for 60 min at room temperature. After blocking endogenous peroxidase activity with 3% H_2_O_2_ for 10 min, the sections were visualized with Vectastain ABC Elite (Vector Laboratories) and 0.5 mg/mL 3, 3,9-diaminobenzidine enhanced with ammonium nickel sulfate, beta-D glucose, ammonium chloride, and beta-glucose oxidase. After dehydrating with graded ethanol and xylene, the sections were mounted using Vector mounting medium.

### Cell counting

Area contours were drawn and measured in every 50^th^ section. The active caspase-3 and AIF-positive cells (DCA = 7, vehicle = 8, 24 h) were counted at 400× magnification in the border zone of the injured cortex within an area of 0.196 mm^2^ (one visual field) using Micro Image (Olympus, Japan). Three sections were counted from each brain with an interval of 250 μm. The average was defined as n = 1 when comparing different brains. All of the counting was carried out by investigators blinded to group assignment.

### Brain injury evaluation

The gray-matter area was determined by measuring the MAP2 immunoreactive area from eight serial sections per animal. Brain injury was evaluated by the volume of infarction and total hemispheric tissue loss, and neuropathological scoring was performed by a person who did not have prior knowledge of the groups. The cerebral subcortical white matter area was determined by measuring the MBP immunoreactive area in five serial sections per animal. The MAP2 or MBP-positive and -negative areas in each section were measured in both hemispheres using Micro Image (Olympus, Japan). The tissue volume was calculated from the MAP2 or MBP-positive or -negative areas according to the Cavalieri principle using the following formula: V = ΣA × P × T, where V = the total volume, ΣA = the sum of area measurements, P = the inverse of the sampling fraction, and T = the section thickness. The total hemispheric tissue loss was calculated as the MAP2-positive volume in the contralateral hemisphere minus the MAP2-positive volume in the ipsilateral hemisphere. The MBP-positive area was measured in each hemisphere, and the total MBP tissue loss ratio was calculated as: ((contralateral hemisphere - ipsilateral hemisphere) / contralateral hemisphere) × 100%. The neuropathological score of gray matter from different brain regions was assessed. Briefly, the cortical injury was graded from 0 to 4 with 0 being no observable injury and 4 indicating confluent infarction. The injury in the hippocampus, striatum, and thalamus was assessed both with respect to hypotrophy (scored from 0 to 3) and injury/infarction (scored from 0 to 3), resulting in a total possible score of 22.

### Sample preparation for Western blot, caspase activity, and ELISA kits

The pups were sacrificed by decapitation at 6 h or 24 h after HI. Tissue from the parietal cortex (including the hippocampus) in each hemisphere was rapidly dissected out on a bed of ice. Tissue samples were homogenized immediately using a 2-mL glass/glass homogenizer (Merck Eurolab, Gothenburg, Sweden) on ice, and isolation buffer was added (15 mM Tris-HCl (pH 7.6), 320 mM sucrose, 1 mM dithiothreitol, 3 mM EDTA-K, and 0.5% protease inhibitor cocktail, which was added fresh immediately prior to use). Half of the homogenates were aliquoted and stored at −80°C, and the other half were centrifuged at 800 × *g* at 4°C for 10 min. The pellet fraction was washed, recentrifuged, and saved as the nuclear fraction. The supernatants were further centrifuged at 9200 × *g* for 15 min at 4°C, producing mitochondrial and synaptosomal fractions in the pellet and crude cytosolic fractions in the supernatants. The enriched mitochondrial fraction was washed and centrifuged. All fractions were stored at −80°C.

### Immunoblotting

Protein concentration was determined using the bicinchoninic acid method. Samples with a volume of 65 μL were mixed with 25 μl NuPAGE LDS 4× sample buffer (ThermoFisher Scientific, NP0007) and 10 μl reducing agent (ThermoFisher Scientific, NP0004) and heated at 70°C for 10 min. Individual samples of 10 μg protein were loaded and run on 4%-12% NuPAGE Bis-Tris gels (Novex) and transferred to reinforced nitrocellulose membranes. After blocking with 30 mM Tris-HCl (pH 7.5), 100 mM NaCl, and 0.1% Tween 20 containing 5% fat-free milk powder for 60 min at room temperature, the membranes were incubated with the following primary antibodies: rabbit anti-Actin (1:200 dilution, Sigma, A2066), goat anti-AIF (1:1000 dilution, Santa Cruz Biotechnologies, sc-9416), rabbit anti-LC3 (1:1000 dilution, Cell Signaling, 2775), and rabbit anti-SQSTM1 (1:1000 dilution, Enzo Life Science, PW9860), rabbit anti-phospho-DRP1 (Ser637) (1:1000 dilution, Cell Signaling, 4867); mouse anti-OPA1 (1:1000 dilution, BD bioscience, 612606), at room temperature for 60 min. After washing, the membranes were incubated with a peroxidase-labeled secondary antibody for 30 min at room temperature (goat anti-rabbit (1:2,000 dilution), horse anti-goat (1:2,000 dilution), or horse anti-mouse (1:4,000 dilution)). Immunoreactive species were visualized using the Super Signal West Dura substrate (ThermoFisher Scientific, 34075) and an LAS 3000 cooled CCD camera (Fujifilm, Japan). Actin was used as the loading control.

### Caspase-3 activity assay

Protein concentration was determined using the bicinchoninic acid method. For the caspase-3 activity assay, samples of 25 μl homogenate were mixed with 75 μl extraction buffer containing 50 mM Tris-HCl (pH 7.3), 100 mM NaCl, 5 mM EDTA, 1 mM EGTA, 1 mM PMSF, and 1% protease inhibitor cocktail on a microtiter plate. After incubation for 15 min at room temperature, 25 μM Caspase-3-substrate (DEVD) in 100 μl extraction buffer, without protease inhibitors or CHAPS but with 4 mM DTT, was added. Caspase-3 activity was measured using a Spectramax Gemini microplate fluorometer (excitation/emission wavelength 380/460 nm every 2 min for 2 h at 37°C) and expressed as pmol AMC released/mg protein per minute.

### PDH assay

The samples of enriched mitochondrial fraction were used for PDH (Biovision, Milpitas, K679-199) activity measurement according to the manufacturer's instructions. Briefly, 5 μl enriched mitochondrial fraction was mixed with 45 μl PDH assay buffer and 50 μl reaction mix, and the absorbance was measured immediately at 450 nm in kinetic mode for 60 min at 37°C. The activity was calculated according to the standard curve and expressed as mU/mg protein.

### Acetyl-coenzyme A assay

AcCoA was measured in the mitochondrial fraction by using an assay kit (Sigma-Aldrich, MAK039) according to the manufacturer's instructions. Briefly, 40 μl sample was mixed with 20 μl 1N perchloric acid and centrifuged at 13,000 × g at 4°C for 10 min to remove insoluble material. The supernatant was neutralized with 3M potassium bicarbonate solution at pH 6.8 and spun down to pellet the potassium bicarbonate. The supernatant sample (20 μl) was mixed with 30 μl AcCoA assay buffer in a 96 well plate and then 10 μl AcCoA quencher was added and incubated for 5 min at room temperature. Finally, 2 μl quench remover was added to each sample well and incubated for an additional 5 min. After adding 50 μl of the reaction mix to each well and incubating the reaction for 10 min at 37°C, the AcCoA concentration was measured in a Spectramax Gemini microplate fluorometer (with an excitation wavelength of 535 nm and an emission wavelength of 587 nm) and expressed as pmol/mg protein.

### Lactate assay

The lactate concentrations in the cortex homogenate samples at 24 h after HI were measured using the Lactate Colorimetric Assay Kit (Biovision, Milpitas, K607-100) according to the manufacturer's instructions. Briefly, samples of homogenate (25 μl) were mixed with 25 μl assay buffer on a microtiter plate and then 50 μl reaction mixtures containing 46 μl lactate assay buffer, 2 μl lactate enzyme mix, and 2 μl probe were added. After incubation for 30 min at room temperature, the lactate concentration was measured with a Spectramax Gemini microplate reader based on the absorbance (OD 570 nm) and expressed as nmol/mg protein.

### Mitochondrial DNA copy number measurement

Total DNA from the cortex was isolated using a genomic DNA isolation kit (DNeasy Blood & Tissue Kit, Qiagen). The amount of mitochondrial DNA relative to nuclear genomic DNA was determined by quantitative real-time PCR. The two nuclear genes were *Ywhaz* (sense: 5′-GAG GAA GAA TCG TGA GTT AGT T-3′, anti-sense: 5′-TGG TGA TGG TTG AGA CAG A-3′) and *36B4* (sense: 5′-GTT GTT AGC CTG TGA TAG CA-3′, anti-sense: 5′-CCGACCAGCAATGTTCTATT-3′), and the two mitochondrial genes were *ND4* (sense: 5′-CCT CAG TTA GCC ACA TAG C-3′, anti-sense: 5′- GAT TCG TTC GTA GTT GGA GTT-3′) and *D-loop* (sense: 5′-GCC CAT GAC CAA CAT AAC TG-3′, anti-sense: 5′-CCT TGA CGG CTA TGT TGA TG-3′). The relative mitochondrial DNA level was calculated based on the threshold cycle (Ct) as 2-Δ (ΔCt).

### Mitochondria biogenesis, fission, and fusion gene mRNA expression

Total RNA was isolated with an RNA isolation kit (RNeasy Mini Kit, Qiagen, 74104) according to the manufacturer's instructions. The concentration and purity of all RNA samples were determined using a Nanodrop spectrophotometer (Nanodrop Technologies). The integrity of the RNA was measured using an Experion RNA StdSens Analysis kit (Bio-Rad) on an Automated Electrophoresis Station machine (Bio-Rad). One microgram of total RNA was reverse transcribed using the QuantiTect Reverse Transcription kit (Qiagen, 205311). Quantitative real time PCR was performed using the LightCycler 480 instrument (Roche Diagnostics, Germany) and the SYBR Green technique according to the manufacturer's instructions. The primers used in the qRT-PCR reactions were designed by Beacon Designer software (free trial, PREMIER Biosoft) and included the mitochondrial biogenesis genes *Pgc-1α* (sense: 5′-CCA GGT CAA GAT CAA GGT-3′, antisense: 5′-CGT GCT CAT AGG CTT CAT A-3′), *Tfam* (sense: 5′-ACC TCG TTC AGC ATA TAA CAT T-3′, anti-sense: 5′-ATC ACT TCG TCC AAC TTC AG-3′), and *Nrf1* (sense: 5′-CCA CAG GAG GTT AAT TCA GAG-3′, antisense: 5′-ACA TCA CTG CGG ACA TTG-3′) and the mitochondrial fission and fusion genes *Drp1* (sense: 5′-TGC TCA GTA TCA GTC TCT TC-3′, antisense: 5′-GGT TCC TTC AAT CGT GTT AC-3′), *Mfn1* (sense: 5′-CAC TGA TGA ACA CGG AGA A-3′, antisense: 5′-CGA CGG ACT TAC AAC CTT-3′), *Opa1* (sense: 5′-CCT GTG AAG TCT GCC AAT-3′, antisense: 5′-TTA GAG AAG AGA ACT GCT GAA AT-3′). The reference genes *Ywhaz* (sense: 5′-CCT CAA CTT CTC TGT GTT CTA TT -3′, antisense: 5′-ACG ACT CTT CAC TTA ATG TAT CAA-3′), *Sdha* (sense: 5′-TTG CCT TGC CAG GAC TTA-3′, antisense: 5′-CAC CTT GAC TGT TGA TGA GAA T-3′) [52]. The relative expression levels of mRNAs were calculated by the method of geometric averaging of multiple internal control genes.

### Statistical analysis

The Statistical Package for the Social Sciences 21.0 (SPSS, IBM, NY, USA) was used for all analyses. Comparisons between groups were performed by Student's *t*-test, and data with unequal variance were compared with the Mann-Whitney *U*-test. Two-way ANOVA followed by a Bonferroni post hoc test was used for multiple comparison correction of data from more than two groups. Results are presented as means ± standard errors of the mean (SEM). *p* < 0.05 was considered statistically significant.
